# The Serum Level of IL-31 in Patients with Chronic Kidney Disease-Associated Pruritus: What Can We Expect?

**DOI:** 10.3390/toxins14030197

**Published:** 2022-03-07

**Authors:** Karolina Świerczyńska, Piotr K. Krajewski, Danuta Nowicka-Suszko, Rafał Białynicki-Birula, Magdalena Krajewska, Jacek C. Szepietowski

**Affiliations:** 1 Department of Dermatology, Venereology and Allergology, Wroclaw Medical University, 50-368 Wroclaw, Poland; karolina.swierczynska@student.umw.edu.pl (K.Ś.); piotr.krajewski@student.umw.edu.pl (P.K.K.); danuta.nowicka-suszko@umw.edu.pl (D.N.-S.); rafal.bialynicki-birula@umw.edu.pl (R.B.-B.); 2 Department of Nephrology and Transplantation Medicine, Wroclaw Medical University, 50-529 Wroclaw, Poland; magdalena.krajewska@umw.edu.pl

**Keywords:** interleukin-31, chronic-kidney-disease-associated pruritus, hemodialysis patients, renal failure

## Abstract

Chronic-kidney-disease-associated pruritus (CKD-aP) is one of the most common and burdensome dermatological symptoms affecting patients undergoing dialysis, and its etiopathogenesis has still not been fully discovered. This study was designed to investigate the possible contribution of interleukin-31 (IL-31) to the pathogenesis of itch in patients undergoing maintenance hemodialysis (HD). We evaluated the serum level of IL-31 in HD patients with pruritus, in HD patients without pruritus and in healthy controls, as well as its correlation to the severity of itch. The study enrolled 175 adult subjects. The participants were divided into three groups. Group A included 64 patients on maintenance HD with CKD-aP, Group B included 62 patients on maintenance HD not reporting CKD-aP and Group C included 49 healthy controls. Pruritus severity was assessed using the Numerical Rating Scale (NRS), and the serum levels of IL-31 were measured. The results showed that the IL-31 serum level was significantly higher in the itchy group (*p* < 0.001) in comparison to the patients free from pruritus. Moreover, a marginal trend towards significance (r = 0.242, *p* = 0.058) was observed between the IL-31 serum level and itch intensity. Our study supports earlier findings on the extended role of IL-31 in the development of CKD-aP.

## 1. Introduction

Chronic-kidney-disease-associated pruritus (CKD-aP) is one of the most common and burdensome dermatological symptoms affecting patients undergoing dialysis. The prevalence of CKD-aP has been very variable over the years, yet according to the most comprehensive observational study of hemodialysis (HD) that included patients from 12 different countries, approximately 40% of patients with end-stage renal disease (ESRD) report moderate-to-severe pruritus [1]. So far, conducted studies have unequivocally proven that this condition not only has a negative impact on sleep, mood, daily activities and quality of life (QoL), but it also increases the mortality risk of HD patients [2,3]. Despite the high international prevalence, this problem seems to be markedly underestimated in clinical practice. The reason for this trend may be the lack of knowledge regarding effective therapies for CKD-aP [4]. Despite the long-standing studies and many suggested theories, the complex etiopathogenesis of CKD-aP has still not been fully discovered. Among the most important factors contributing to the development of CKD-aP, the following ones can be mentioned: uremic toxins (UTs), immune dysfunction, altered opioid transmission, skin dryness (xerosis), neuropathy, dialysis modality and its parameters [5]. Thereby, gold-standard therapy in this condition remains unclearly defined, and determining an effective clinical approach is still a challenge for dermatologists and nephrologists. CKD-aP is often recurrent and does not respond to available therapeutic methods. Further research in the field of the pathophysiology of CKD-aP may lead to a revolution in the therapeutic management of HD patients suffering from pruritus.

Reduced renal function in patients with chronic kidney disease (CKD) precludes the proper elimination of various metabolites, which are known as UTs. Recently, more and more studies indicate the key role of UTs in the progression of CKD and its wide range of severe complications. In the list of medium molecules classified as UTs, a few interleukins can be found, namely interleukin-10, -18, -1*β* and -6 [6]. Therefore, the role of the immune system seems to play a vital role in CKD-aP. Some studies point out a deranged balance of T helper (TH) cell differentiation towards Th1 predominance in patients with CKD-aP, which allows one to consider this condition as a systemic inflammatory phenomenon. This hypothesis was supported by the elevated serum levels of the C-reactive protein (CRP) and the inflammatory cytokines interleukin (IL)-2 and IL-6 in HD patients with pruritus versus those free from pruritus [7,8].

Interleukin-31 (IL-31) is a T-cell-derived cytokine that takes part in the symptomatology of pruritus, and both IL-31 and its receptor have become potential therapeutic targets for a range of pruritic disorders [9]. It was found that IL-31 signaling through a heterodimeric receptor complex composed of an IL-31Rα subunit and an oncostatin M receptor β subunit (OSMRβ), expressing on the keratinocytes and the epithelial cells, induces severe dermatitis and pruritus in transgenic mice [10]. Moreover, the serum level of IL-31 was elevated in patients with atopic dermatitis. Performed studies revealed a positive correlation between the IL-31 serum level and the Scoring Atopic Dermatitis Index (SCORAD) and also between IL-31 levels and the severity of pruritus-[11,12,13]. Similar findings were reported in clinical studies concerning other pruritic dermatological diseases, such as prurigo nodularis and psoriasis [14,15]. Although the majority of so-far published studies reported an increased serum IL-31 level in patients suffering from CKD-aP, the correlation between IL-31 and itch intensity is still not clear [16,17,18,19]. Therefore, this study was designed to investigate the possible contribution of IL-31 to the pathogenesis of itch in patients undergoing maintenance HD. We aimed not only to evaluate the serum level of IL-31 in HD patients with pruritus, in HD patients without pruritus and in healthy controls, but also to correlate the serum level of IL-31 with itch severity. To evaluate the characteristics and the intensity of the itch, the Numerical Rating Scale (NRS) and a new instrument—the Uraemic Pruritus in Dialysis Patient (UP-Dial) questionnaire—were used.

## 2. Results

**Figure 1:**
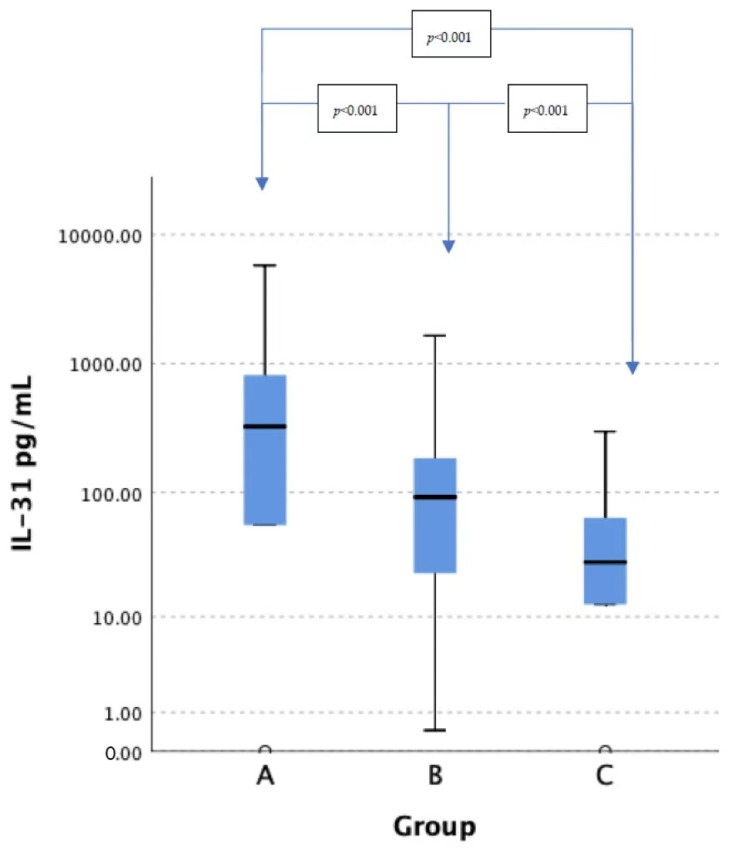
Serum level of IL-31 in study groups. IL-31—interleukin-31, Group A—patients with ESRD on maintenance HD reporting CKD-aP, Group B—patients with ESRD on maintenance HD not reporting CKD-aP, Group C—healthy controls.

In the group of HD patients suffering from CKD-aP, 50% were males, and the average age was 61.1 ± 15.9 years. The mean NRS score was 4.9 ± 2.2 points. According to the NRS cut-offs, mild pruritus was reported in 14.5% of the cases, moderate in 59.7%, severe in 22.6% and very severe in 3.2%. The mean UP-Dial score was 14.2 ± 9.8 points. In 58% of the CKD-aP patients, itch interfered with their sleep, and only 29% did not report the influence of itch on the following activities: work or study, social interaction, mood or any sexual activities. The mean serum level of IL-31 was 679.9 ± 1112.3 pg/mL in a group of HD patients reporting CKD-aP, 176.1 ± 290.7 pg/mL in a group of HD patients not suffering from CKD-aP and 57.3 ± 65.1 pg/mL in a group of healthy controls (Figure 1). The IL-31 serum level was significantly higher in the itchy group (*p* < 0.001) in comparison to the patients free from pruritus. Moreover, there was a significant difference (*p* < 0.001) in IL-31 serum levels between HD patients with and without pruritus and healthy controls (*p* < 0.001 and *p* = 0.019, respectively).

**Figure 2:**
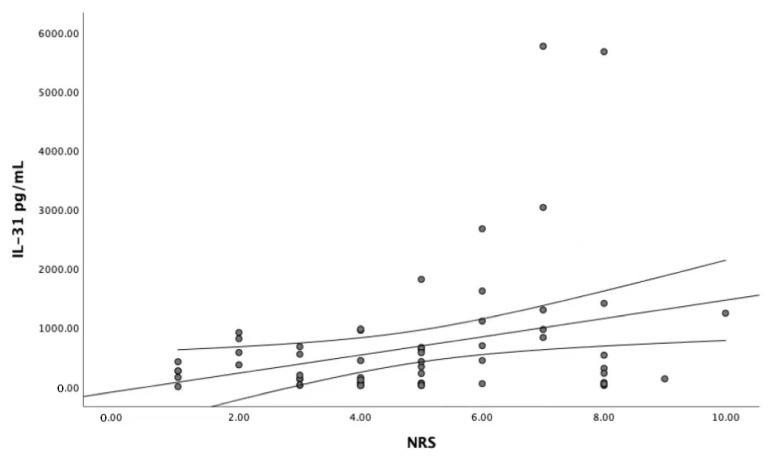
Dependence between IL-31 serum levels and NRS score (r = 0.242, *p* = 0.058). NRS—Numerical Rating Scale, IL-31—interleukin-31, circles—the serum level of interleukin-31.

Despite the above-mentioned results, a marginal trend towards significance (r = 0.242, *p* = 0.058) was observed between the IL-31 serum level and the worst itch intensity during the last 3 days as assessed by the NRS (Figure 2).

The UP-Dial questionnaire total score strongly correlated with the NRS (r = 0.399, *p* = 0.001). Additionally, each domain of the UP-Dial showed a significant correlation with the NRS scores (detailed data not shown). However, the correlation between the serum level of the IL-31 and the UP-Dial total score was not statistically significant.

## 3. Discussion

CKD-aP is a prevalent and very burdensome comorbidity associated with CKD, and its pathogenesis is not fully understood. A steering committee of patients, caregivers, researchers and clinicians in Canada that is assembling a list of the top-10 research priorities for kidney disease agreed that determining the causes, effective treatments and preventative measures for CKD-aP is essential for the holistic management of HD patients [20]. This study underlined that pruritus is a relevant symptom from which patients with CKD suffer, and that its effective identification and treatment still causes many clinical issues [21]. Therefore, the importance of further research in the field of the pathogenesis of CKD-aP is undeniable, and their results may provide more effective and innovative management options for HD patients with this condition.

So far, only a few studies evaluating the contribution of IL-31 in the pathogenesis of CKD-aP have been published, and their results were ambiguous. In this study, which aimed to deepen the immune aspect of CKD-aP pathogenesis, the results showed a significant elevation of IL-31 in patients reporting CKD-aP compared to HD patients without pruritus. These findings correspond with Ko et al.’s [16] study, which pointed to an extended role of IL-31 in pruritic skin disease by showing that IL-31 levels were significantly higher in patients with pruritus symptoms than in those without. Moreover, in a cross-sectional study on 65 HD patients and 49 healthy controls, Oweis et al. [17] showed similar findings to ours. The IL-31 serum level was significantly higher in the HD patients than in the control group, while the differences between the levels of IL-13 and IL-33 in these groups were not statistically significant. Interestingly, a study performed by Haggag et al. [18] showed an apparently increased level of IL-31 in CKD-aP patients; however, the difference between the itchy and non-itchy group did not reach statistical significance. Finally, a study performed on 145 participants, which consisted of HD patients, peritoneal dialysis (PD) patients, kidney transplant patients and healthy controls, showed no significant differences in serum IL-31 levels among the study groups [19,22]. Notwithstanding, they concluded that IL-31 might play a role in the development of longitudinal nail ridges by revealing significantly higher IL-31 levels in a group of patients with this nail condition.

Otherwise, Ko et al. [16] additionally demonstrated a positive relationship between IL-31 levels and the Visual Analogue Scale (VAS) score of pruritus intensity. This result is not fully analogous to our research, as we did not prove a significant correlation between the IL-31 serum levels and the pruritus intensity assessed with the NRS. Nevertheless, this dependency reached a borderline level of statistical significance (*p* = 0.058). The reason for this discrepancy may be that the present study did not have a big enough study group. Oweis et al. [17] also failed to indicate the dependence between IL-31 and itch severity. However, the serum level of IL-13 showed a statistically significant relationship with the severity of itch, as measured by the PGS (pruritus grading system). Moreover, in the present study, the correlation between IL-31 and the UP-Dial questionnaire was also not achieved. This tool, although allowing for a multivariate assessment of pruritus, is not widely used and is only available in a few languages; therefore, the best instrument to assess pruritus with reliable results is the NRS. Furthermore, the correlation between the age of the patients and the serum level of IL-31 was not revealed. Additionally, in published studies concerning the level of IL-31 in different conditions, e.g., Hodgkin lymphoma, the same phenomenon was observed [23].

Despite some inaccuracies in the recently carried-out research, the trend of the higher levels of IL-31 in patients experiencing CKD-aP is noticeable. These findings indicate a possible role of the dysregulation of the immune system in the pathogenesis of CKD-aP in HD patients. Kimmel et al. [8] strongly support this theory, indicating the elevated pro-inflammatory cytokines and the serum acute phase proteins as well as the predominant differentiation of the Th1 cells in HD patients with CKD-aP. The performed studies showed an increased level of CRP, IL-6 and IL-2 in patients with CKD-aP versus patients without it [7,8,22]. However, taking into consideration that IL-31 is expressed primarily in the Th2 cells, the results of our study may not be in accordance with findings about the predominant differentiation of the Th1 cells in CKD-aP patients.

IL-31 is a novel cytokine that has a proven role in the pathogenesis of skin inflammation, airway hypersensitivity and in a range of pruritic diseases [9]. Studies reported not only its higher serum level in conditions such as atopic dermatitis, psoriasis, cutaneous T-cell lymphoma and allergic contact dermatitis, but also its increased level in skin samples from patients with prurigo nodularis, dermatomyositis or chronic urticaria [11,12,13,14,24,25,26]. Likewise, our team investigated the expression of IL-31 in the skin of patients with CKD-aP in comparison to patients free from itching in CKD [27]. In patients with CKD-aP, IL-31 expression was significantly more common across the whole epidermis than limited to the suprabasal layers of the epidermis. However, there was no significant difference in the overall IL-31 expression between patients with and without pruritus. It should be considered that this preliminary study was limited by the number of subjects—40 patients. To the best of our knowledge, until now, more extended studies in this area have not been performed.

Moreover, therapies targeting IL-31 reduced itch successfully. Grimstad et al. [28] demonstrated that the IL-31 antibody ameliorates scratching behavior in an atopic dermatitis-like murine model during the onset of clinical skin manifestations. To date, in clinical practice, only the anti-IL-31 RA antibody nemolizumab reached phase III of the clinical trial in patients with atopic dermatitis, which, combined with topical agents, resulted in a greater reduction in pruritus [29]. Similar effects of nemolizumab were found in a group of patients with prurigo nodularis [30]. Unfortunately, until now, a reduction in itch intensity in CKD-aP patients after the administration of nemolizumab was not proven [31]. However, this study was limited by a very specific Japanese population and small subgroups receiving different doses of the drug. Further investigations with bigger, more representative groups are required. Therefore, another ongoing, multicenter, double-blind, placebo-controlled study evaluating the efficacy of nemolizumab in CKD-aP patients may verify these results [32]. Nevertheless, it seems that overexpressed IL-31 may accelerate to CKD-aP through the activation of peripheral sensory nerves, as the expression of the IL-31Rα subunit was documented in a subpopulation of the TRPV1+/TRPA1+ neurons of the dorsal root ganglia in mice [5].

This study showed some limitations. We did not investigate the possible contribution of other inflammatory interleukins in CKD-aP etiopathogenesis. Further studies in this area are necessary. Moreover, the possible correlation between itch intensity and the removal of the IL-31 molecule during the dialysis session should be estimated. The IL-31 with the size of 18 kDa is classified as a middle molecule, and its clearance using high-flux membranes is low [33]. Therefore, the measurement of the IL-31 levels after the hemodialysis session and the comparison of this result with itch intensity may lead to an interesting outcome.

## 4. Conclusions

CKD-aP is a pervasive and multifactorial condition strongly affecting patients’ quality of life. Its etiopathogenesis involves multiple mediators and remains not fully understood. This study revealed a significantly higher serum level of IL-31 in HD patients with CKD-aP versus HD patients without CKD-aP. This tendency supports earlier findings about the extended role of this interleukin in the development of CKD-aP. It is hoped that further research targeting this cytokine may bring more information about the immunological aspect of CKD-aP etiopathogenesis and possible effective and innovative management options for HD patients with CKD-aP.

## 5. Material and Methods

### 5.1. Study Population

This study enrolled 175 adult subjects of which 85 (48.6%) were females and 90 (51.4%) were males. The mean age of the study population was 58.46 ± 15.8 years. The participants were divided into three groups. Group A included 64 patients with ESRD on maintenance HD with CKD-aP, Group B included 62 patients with ESRD on maintenance HD not reporting CKD-aP and Group C included 49 healthy controls who corresponded with age and gender to the patients from the study group and had no history of any pruritic or systemic diseases. The exclusion criteria for this study was as follows: primary skin disorders, other itchy skin diseases, psychiatric disorder or communication problems, age under 18 years, antipruritic therapy and patients’ refusal. All participants underwent a physical and dermatological examination. Medical history, including cause of renal failure, duration of HD and previous treatment of pruritus was taken. Most of the patients received dialysis 3 times a week; only 8 of them received dialysis 2 times a week. The average dialysis vintage was 48.8 ± 51.9 months, and the hemodialysis was performed using bicarbonate dialysate. All individuals were undergoing hemodialysis using high-flux polysulfone membrane dialyzers. The single pool Kt/V accessing the dialysis adequacy for the whole study population was 1.28 (SD ± 0.39). In a group of patients reporting CKD-aP, it was 1.21 (SD ± 0.31), and in a group of patients not reporting CKD-aP, it was 1.34 (SD ± 0.43). The difference between these two groups was not statistically significant. Therefore, the level of hemodialysis adequacy did not have an impact on itch sensation.

The most common cause of end-stage renal disease in the entire study cohort was glomerulonephritis (19.8%); in Group A it was diabetic kidney disease (17.7%) and in Group B it was also glomerulonephritis (26.6%). Substantively, the most frequent cause of kidney disease in the general population is diabetic nephropathy. However, currently published studies emphasize that the cause of kidney failure does not determine CKD-aP [34,35].

The average dialysis vintage was 48.8 ± 51.9 months. More than half of the HD patients underwent dialysis through an arterio-venous fistulae (65.1%) and the rest through a tunneled internal jugular central venous catheter. Table 1 summarizes the baseline characteristics of the participants.

This research obtained ethical approval by the Wroclaw Medical University Ethics Committee (Consent no. 26/202, date: 29 January 2021). Patients were included in the research after obtaining their informed consent.

### 5.2. Pruritus Assessment

Pruritus severity was assessed using the NRS, which is an easily accessible instrument commonly used to evaluate the intensity of pruritus. On a scale from 0 to 10 points, participants indicated the worst itch intensity they experienced during the last 3 days. Patients were classified according to the severity of pruritus as follows: NRS < 3—mild pruritus, NRS > 3 and < 7—moderate pruritus, NRS > 7 and < 9—severe pruritus and NRS > 9—very severe pruritus [36]. Moreover, subjects completed a Polish, validated version of the UP-Dial questionnaire [37]. This 14-item instrument was designed to characterize itch in dialysis patients. It evaluates three dimensions of CKD-aP: signs and symptoms, sleep and psychosocial burden during the last 2 weeks. These domains investigate all of the important aspects of chronic itch: frequency, intensity, distribution, skin lesions caused by itch, impact on sleep and the psychosocial status of the patient [38]. The total score of the UP-Dial also enables one to classify the severity of the itch.

### 5.3. Samples and Interleukin-31 Serum Level Measurement

Blood samples of 9 mL were collected from all of the participants. Blood was drawn during the routine drawing-of-blood from an arterio-venous fistula or from a permanent HD catheter just before starting a dialysis session in dialysis patients. Subsequently, the samples were centrifuged at 3000× *g* rpm for 15 min. The received serum was stored at—80 °C until the next laboratory steps were performed. The serum level of IL-31 was subsequently measured by the ELISA (enzyme-linked immunosorbent assay) technique using the Nori Human IL-31 ELISA Kit (catalog number: GR 111374, GENORISE SCIENTIFIC, Inc., Glen Mills, PA, USA), according to the manufacturer’s instructions. The absorbance of the sample was measured at 450 nm using the EPOCH (BioTEK^®^ Instruments, Inc., Winooski, VT, USA) adjustable microplate reader. IL-31 had a test range of 50–3200 pg/mL and a sensitivity of 10 pg/mL.

### 5.4. Statistical Analysis

A statistical analysis of the obtained results was performed with the use of the IBM SPSS Statistics v. 26 (SPSS Inc., Chicago, IL, USA) software. All data were assessed for normal or abnormal distribution. The minimum, maximum, mean and standard deviation were calculated. Quantitative variables were evaluated using the Mann–Whitney U test and Spearman’s or Pearson’s correlations. For the qualitative data, the chi-squared test was used. For differences in more than two groups, the Kruskal–Wallis one-way analysis of variance on ranks was performed. A two-sided *p* of a value lower than 0.05 was considered significant.

## Figures and Tables

**Table 1:** Baseline characteristics for hemodialysis patients and controls.

Variable	Group A, N (%)	Group B, N (%)	Group C, N (%)
Age, mean (±SD)	61.1(SD ± 15.9)	63.9 (SD ± 15.6)	48.0 (SD ± 10.24)
Gender: Male	31 (50.0%)	30 (46.9%)	24 (48.9%)
Female	31(50.0%)	34 (53.1%)	25(51.0%)

Group A—patients with ESRD on maintenance HD reporting CKD-aP. Group B—patients with ESRD on maintenance HD not reporting CKD-aP, Group C—healthy controls.

## Data Availability

The datasets generated and analyzed in the current study are available from the corresponding author on reasonable request.
